# QEEG characteristics and spectrum weighted frequency for children diagnosed as autistic spectrum disorder

**DOI:** 10.1186/1753-4631-4-4

**Published:** 2010-09-30

**Authors:** Nada Pop-Jordanova, Tatjana Zorcec, Aneta Demerdzieva, Zoran Gucev

**Affiliations:** 1 Pediatric Clinic, Faculty of Medicine, University of Skopje, Macedonia

## Abstract

**Background:**

Autistic spectrum disorders are a group of neurological and developmental disorders associated with social, communication, sensory, behavioral and cognitive impairments, as well as restricted, repetitive patterns of behavior, activities, or interests.

The aim of this study was a) to analyze QEEG findings of autistic patients and to compare the results with data base; and b) to introduce the calculation of spectrum weighted frequency (brain rate) as an indicator of general mental arousal in these patients.

**Results:**

Results for Q-EEG shows generally increased delta-theta activity in frontal region of the brain. Changes in QEEG pattern appeared to be in a non-linear correlation with maturational processes.

Brain rate measured in CZ shows slow brain activity (5. 86) which is significantly lower than normal and corresponds to low general mental arousal.

Recent research has shown that autistic disorders have as their basis disturbances of neural connectivity. Neurofeedback seems capable of remediating such disturbances when these data are considered as part of treatment planning.

**Conclusions:**

Prognosis of this pervasive disorder depends on the intellectual abilities: the better intellectual functioning, the possibilities for life adaptation are higher

QEEG shows generally increased delta-theta activity in frontal region of the brain which is related to poor cognitive abilities.

Brain rate measured in CZ shows slow brain activity related to under arousal.

Pharmacotherapy combined with behavior therapy, social support and especially neurofeedback technique promise slight improvements

## Background

Autistic spectrum disorders are a group of neurological and developmental disorders associated with social, communication, sensory, behavioral and cognitive impairments, as well as restricted, repetitive patterns of behavior, activities, or interests. Autistic spectrum disorders (ASD) include autism, pervasive developmental disorder and Asperger's. These are life-changing, family-altering conditions usually diagnosed in early childhood. In recent years, this group of impairment has shown a dramatic increase in prevalence, which raises the clinical and scientific interest worldwide. Main clinical signs of autism as described by Kanner and Asperger in the forties of the last century are still not changed [1-7].

The broad variation in phenotypes and severities within autism spectrum disorders suggests the involvement of multiple predisposing factors, interacting in complex ways with normal developmental courses and gradients. ASD is generally accepted as the most genetic of all the developmental neuropsychiatric syndromes. However, despite more than several decades of genetic study, the precise etiology of ASD still remains unknown, largely due to the genetic and phenotypic diversity, or heterogeneity of this disorder, and the lack of biologically based classification systems [8-10]

Review of some major textbooks on ASD and of papers published between 1961 and 2003 yields convincing evidence for multiple interacting genetic factors as the main causative determinants of autism [11-13].

Epidemiologic studies indicate that environmental factors such as toxic exposures, teratogens, perinatal insults, and prenatal infections such as rubella and cytomegalovirus account only for few cases. These studies fail to confirm that immunizations with the measles-mumps-rubella vaccine are responsible for the surge in autism.

Epilepsy, the medical condition most highly associated with ASD, has equally complex genetic/no genetic (but mostly unknown) causes. Autistic symptoms are frequent in tuberous sclerosis complex and fragile × syndrome, but these disorders account for a small minority of cases. Currently, diagnosable medical conditions, cytogenetic abnormalities, and single-gene defects (e.g. tuberous sclerosis complex, fragile × syndrome, and other rare diseases) together account for <10% of cases [14].

ASD discussed in this article corresponds to pervasive developmental disorder (PDD) of the Diagnostic and Statistical Manual of Mental Disorders, fourth revised edition (DSM-IV) and International Classification of Diseases, tenth revision (ICD-10) [15,16]. As mentioned, as ASD we mean the wide spectrum of developmental disorders characterized by impairments in three behavioral domains: 1) social interaction; 2) language, communication, and imaginative play; and 3) range of interests and activities.

Most pediatricians will have children with this disorder in their practices. They must suspect ASD expeditiously before 17^th ^months of life and diagnosed it around the third year, because that early intervention increases its effectiveness.

An astonishing 556% reported increase in pediatric prevalence between 1991 and 1997, a prevalence higher than that of spina bifida, cancer, or Down syndrome, is probably attributable to heightened awareness and changing diagnostic criteria rather than to new environmental influences [17].

A review of prevalence survey research across the United States and the United Kingdom reported rates of ASD substantially increased from prior surveys indicating 5 to 10 per 10,000 children to as high as 50 to 80 per 10,000 (equivalent to a range of 1 in 200 to 1 in 125 children with ASD) [18]. Another review of research on the epidemiology of autism [courchesne19,] indicated that approximately 60 per 10,000 children (equivalent to a range of 1 in 166 children) are diagnosed with Autistic Spectrum Disorder.

In the region of R. Macedonia ASD also become the most common neurodevelopmental disorder in young children. In our clinical practice, the last five years we have registered the incidence about 1:300 of children treated at the Department for Psychophysiology (the total number of patients is about 900 per year).

The aim of this study was a) to analyze QEEG findings of autistic patients and to compare the results with data base; and b) to introduce the calculation of spectrum weighted frequency - brain rate parameter as an indicator of general mental arousal in these patients.

## Methodology

The diagnosis is made according two statistic manuals (DMS-IV-R and ICD-10). In addition, we applied Autism Diagnostic Observation Schedule (ADOS) comprising observations evaluating language, behavior, and development, some neurological tests, and structured interviews with parents.

Medical history, neuropsychological assessment, biochemical analysis as well as MRI have been realized in all children. Finally, the blood sample for precise genetic assessment is transported to a specific UK laboratory (results are still unavailable).

Quantitative electroencephalographic (QEEG) evaluation or mapping is an assessment instrument designed to pinpoint anomalies in brain function [20-25]. QEEG maps, collected using 19 electrodes based on the International 10-20 system, are quantitative analyses of EEG characteristics of frequency, amplitude and coherence during various conditions or tasks. These data were than statistically compared to an age-matched normative database to reveal a profile of abnormalities [26].

**Figure 1:**
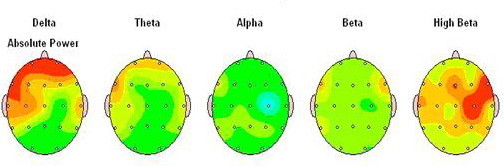
**Summary information for main brain waves in autistic child**.

On the Figure 1 main general characteristics of brain mapping together with z-scores in autistic child is displayed. It is clear that absolute power for delta and theta are increased, but also the power of beta waves due to high anxiety can be increased in some cases. It is very important to know that generally, alpha brain waves are positively related to cortical information processing i.e. cognitive abilities. Alpha and theta brain waves change with age in a non-linear and opposite way. Children with neurological and developmental disorders such as ASD show significantly more delta and theta but less alpha power which correspond with their cognitive impairment [27].

## Results and discussion

### (1) QEEG spectra

The sample comprised 9 autistic children. The mean age of our patients when QEEG was recorded was 4,92 years ± 1,37; while the mean age when diagnosed was 3,5 ± 0,89 years; all patients were boys, and were first and unique children in the family. The mean age of parent was 27, 5 ± 1, 45 years. Minimal educational level of the parents was gymnasium. The socioeconomic level belongs to high middle class.

Clinical evaluation together with Autism Diagnostic Observation Schedule (ADOS) confirmed ASD.

**Figure 2:**
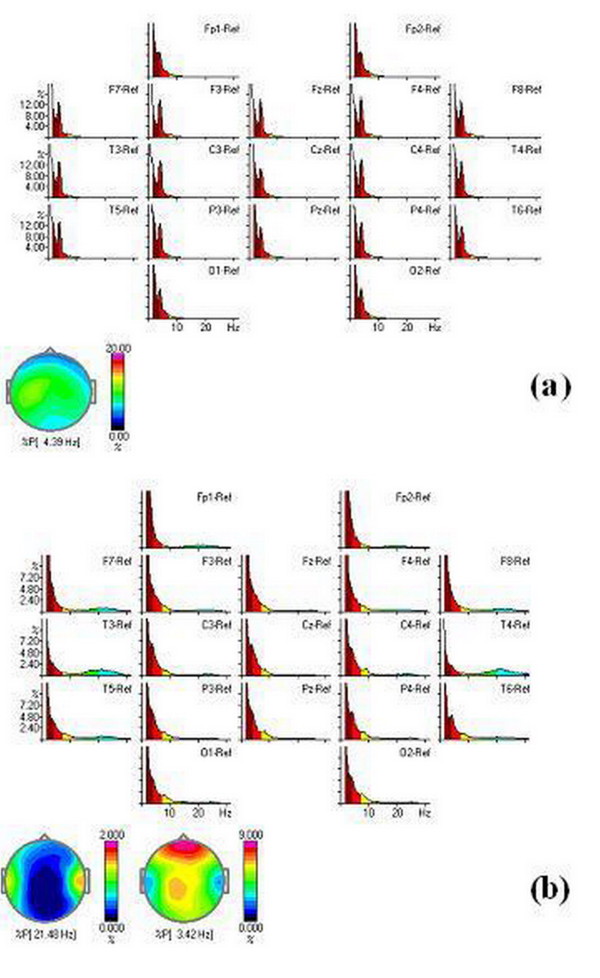
**Spectra mapping of a 3.5 years boys in (a) EC and (b) EO conditions**.

The QEEG recording of autistic children is very difficult due to their hyperactivity, refusal of any sensory contact, and lower intellectual abilities. For QEEG recording patients must be awake and know to open and close their eyes. On Figure 2. spectra for our 3, 5 years autistic boys in EC and EO condition are presented.

It is clear that the dominant power in EC is 4. 39 Hz which belong to low theta brain waves. When the child opens the eyes, slow activity did not change in the frontal and central region, while some high beta activity (21, 48 Hz) appeared especially in centro-temporal regions.

**Figure 3:**
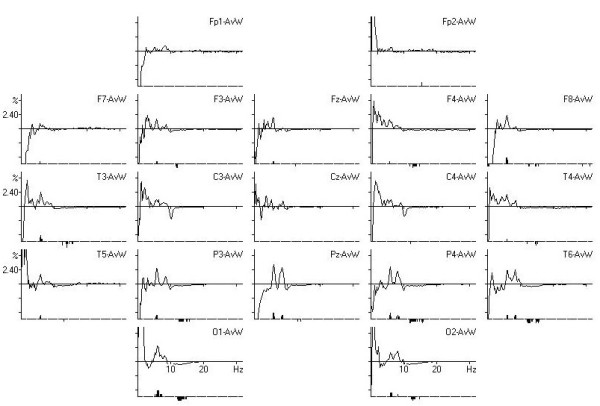
**Comparison of QEEG spectra with data base (Significant difference is marked below the spectra)**.

On Figure. 3 comparison of the QEEG spectra of our boy with normative data base for the same age and gender is displayed. It is clear that significant differences are marked in the theta band.

Concerning QEEG results, Michael Linden, 2005 [28] has identified 4 subtypes of autism. They are:

(1) over focused/over aroused pattern (high beta

(2) abnormal EEG/seizure pattern

(3) high delta/theta

(4) low voltage/metabolic.

Our findings of QEEG belong to the third sub group - high delta/theta.

In the recent research literature on autistic spectrum disorders, some areas of the brain are repeatedly found to differ when compared to normal subjects. Most of these areas are connected to what is called the mirror-neuron system. Mirror neurons are groups of neurons that fire when a person is watching and mentally mirroring the actions of another person. Young children learn to mirror and reflect the behavior and feelings of others, starting with their mother. This mirroring system is crucial for the young child in order to understand the intentions and meanings of other people, as expressed through nonverbal communication. In children with autism, this mirror neuron system is not functioning normally [29].

Six main areas of dysfunction in autism that can be identified using the QEEG: are: (1) amygdale with connections to the orbital and medial frontal areas of the brain, (2) the fusiform gyres, (3) superior temporal gyres with the auditory cortex in the temporal lobe, (4) the anterior insula and the anterior cingulated - both parts of the limbic system (the emotional brain), (5) frontal and parietal-temporal mirror neuron areas, and (6) the prefrontal cortex [30].

What is of even more interest is that, once irregularities in functioning are identified, the child can do EEG biofeedback training using a brain-computer interface that seeks to normalize the brain wave patterns [30-34]. The child watches a game-like display that only moves when they produce the correct patterns. With enough practice, the brain learns these healthy patterns and, as the new, more normal patterns become established, the child's behavior also changes. Unfortunately, our team at the psychophysiology department, we have only starting experience using neurofeedback in our ASD patients.

In some previous EEG/QEEG analyses, maturational delay, including excess slow wave activity, greater coherence and less asymmetry were typical findings [31]; as well as signs of diminished hemispheric lateralization together with excessive bilateral frontal alpha activity [34]. Dawson et al. (1982)[35] published signs of reversed lateralization in 30% autistic patients, while Hashimoto et al. (2001)[36] found spike discharges frequent in frontal regions.

MRI/PET findings in autistic patients confirmed reduction in total grey matter volume, increase in CSF volume, reduced connectivity in fronto-striatal and parietal networks [37], while Bodhaert, et al. (2002) [38] found hypo perfusion of bilateral temporal, superior temporal gyrus, superior temporal sulks in 77%. Murphy et al. (2002) [39] described significant prefrontal hypo metabolism on MRI spectroscopy related to Asperger symptom severity. In our patients no significant changes on MRI have been noticed.

Concerning connectivity, main findings show diminished connectivity in language areas during sentence comprehension and frontal lobe hyper connectivity as well as frontal to other hypo connectivity [19,40-42].

### (2) Brain rate assessment

In addition to the analysis of QEEG characteristics of autistic children we introduced the calculation of spectrum weighted frequency or brain rate as an indicator of general mental activity.

As shown in our previous publications, the brain rate (EEG spectrum weighted frequency) can be considered as an integral state attribute correlated to brain electric, mental and metabolic activity. In particular, it can serve as a preliminary diagnostic indicator of general mental activation (i.e. consciousness level), in addition to heart rate, blood pressure or temperature as standard indicators of general bodily activation [43]. It was shown that bran rate can be used to discriminate between the groups of under-arousal and over-arousal disorders, to assess the quality of sleep, as well as to indicate the IQ changes caused by some environmental toxins [44]. Brain rate is also suitable to reveal the patterns of sensitivity/rigidity of EEG spectrum, including frequency bands related to permeability of corresponding neuronal circuits, based on which the individually adapted neurofeedback protocols can be elaborated [45].

Brain rate is calculated by following formula:

$$f_{b}=\sum_{i}f_{i}P_{i}=\sum_{i}f_{i}\frac{V_{i}}{V}\;\text{with}\;V=\sum_{i}V_{i}$$

where the index *i *denotes the frequency band (for delta *i *= 1, for theta *i *= 2, etc.) and *V_i _*is the corresponding mean amplitude of the electric potential or power. Following the standard five-band classification, one has *f_i _*= 2, 6, 10, 14 and 18, respectively.

In this context, brain rate can serve as an indicator of total brain activity in autistic patients, as well.

**Figure 4:**
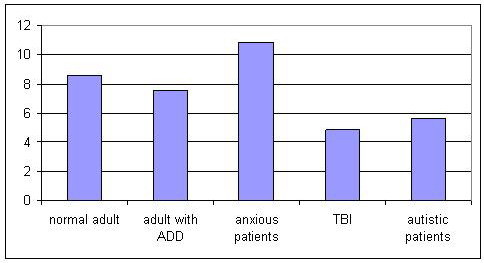
**Comparison of brain rate in different clinical conditions in EO recording**.

Calculated brain rate for autistic children in EO condition was 5, 68. On Figure. 4 brain rate in different clinical conditions at CZ is presented.

It is clear that brain rate as an indicator of general mental activation/arousal is the lowest in patients with traumatic brain injury, followed by brain rate in autistic children and the highest in anxious patients.

**Figure 5:**
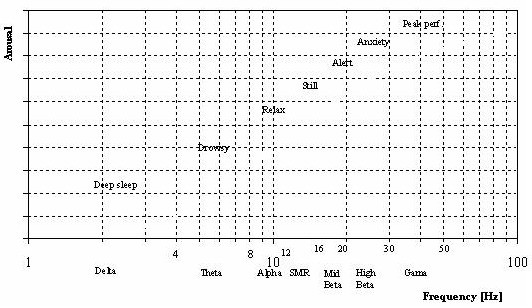
**Standard classification of dominant EEG activity and the correlated mental states i.e. arousal**. Note log scale on the abscissa and absence of scale on the ordinate.

The qualitative dependence of mental arousal on EEG frequency in normal people is summarized in Figure 5.

The obtained results for ANOVA did not showed significant differences for brain rate in various points of QEEG (Table 1). It means that generally brain rate is diminished in all regions.

**Table 1:** Analysis of Variance (new2.sta) (Marked effects are significant at p < .05)

df	MS	SS	df	MS		
**effect**	**effect**	**error**	**error**	**error**	**F**	**p**

1	.05	18. 75	3	6,25	.008	.93

We showed through obtained results that main characteristics of QEEG in autistic children are slowing of the brain activity which is statistically significant when comparing with the data base. These findings are not linear to the maturation of CNS. In addition, calculated brain rate showed lower general mental arousal.

Concerning the treatment, our team uses behavior therapy, medication (psycho stimulants in the morning and neuroleptics in the evening) and in some cases neurofeedback. The last is depending on the intellectual abilities as well as the clinical presentation of the disorder.

## Conclusion

• Prognosis of this pervasive disorder depends on the intellectual abilities: the better intellectual functioning, the possibilities for life adaptation are higher

• QEEG shows generally increased delta-theta activity in frontal region of the brain

• Brain rate measured in CZ shows slow brain activity

• Pharmacotherapy combined with behavior therapy, social support and especially neurofeedback technique promise slight improvements.

## Competing interests

The authors declare that they have no competing interests.

## Authors' contributions

NPJ have made substantial contribution to conception and design, as well as in the acquisition, analysis and interpretation of data. She wrote the article. ZT and DA recorded QEEG, made the analysis and statistics. ZG made important suggestions and found publications about genetics in autistic spectrum disorders and contributed in the writing of the article. All authors have participated sufficiently in the work to take public responsibility for appropriate portions of the content
